# Diet before and during Pregnancy and Offspring Health: The Importance of Animal Models and What Can Be Learned from Them

**DOI:** 10.3390/ijerph13060586

**Published:** 2016-06-14

**Authors:** Pascale Chavatte-Palmer, Anne Tarrade, Delphine Rousseau-Ralliard

**Affiliations:** 1 UMR BDR, INRA, ENVA, Université Paris Saclay, Jouy en Josas 78350, France; anne.tarrade@jouy.inra.fr (A.T.); delphine.rousseau@jouy.inra.fr (D.R.-R.); 2 PremUp foundation, Paris 75006, France

**Keywords:** DOHaD, fetal programming, rodent and non-rodent animal models, 1000 days

## Abstract

This review article outlines epidemiologic studies that support the hypothesis that maternal environment (including early nutrition) plays a seminal role in determining the offspring’s long-term health and metabolism, known as the concept of Developmental Origins of Health and Diseases (DOHaD). In this context, current concerns are particularly focused on the increased incidence of obesity and diabetes, particularly in youth and women of child-bearing age. We summarize key similarities, differences and limitations of various animal models used to study fetal programming, with a particular focus on placentation, which is critical for translating animal findings to humans. This review will assist researchers and their scientific audience in recognizing the pros and cons of various rodent and non-rodent animal models used to understand mechanisms involved in fetal programming. Knowledge gained will lead to improved translation of proposed interventional therapies before they can be implemented in humans. Although rodents are essential for fundamental exploration of biological processes, other species such as rabbits and other domestic animals offer more tissue-specific physiological (rabbit placenta) or physical (ovine maternal and lamb birth weight) resemblances to humans. We highlight the important maternal, placental, and fetal/neonatal characteristics that contribute to developmentally programmed diseases, specifically in offspring that were affected *in utero* by undernutrition, overnutrition or maternal diabetes. Selected interventions aimed at prevention are summarized with a specific focus on the 1000 days initiative in humans, and maternal exercise or modification of the n-3/n-6 polyunsaturated fatty acid (PUFA) balance in the diet, which are currently being successfully tested in animal models to correct or reduce adverse prenatal programming. Animal models are essential to understand mechanisms involved in fetal programming and in order to propose interventional therapies before they can be implemented in humans. Non-rodent animals are particularly important and should not be neglected, as they are often more physiologically-appropriate models to mimic the human situation.

## 1. Developmental Origins of Health and Diseases

Although earlier works suggested that developmental conditions during pregnancy were likely to impact postnatal development up to adulthood, Barker and colleagues’ epidemiological studies were the first to clearly demonstrate the association between low birth weight, used as a proxy for impaired fetal nutrition, and increased risks of non-communicable diseases such as diabetes and hypertension [1,2]. “Fetal programming”, now currently referred to as the Developmental Origins of Health and Disease (DOHaD), was subsequently demonstrated to be the consequence of environmentally-induced perturbations during development, affecting not only those small for gestational age but also those large for gestational age infants. The prevalence of metabolic disorders has increased considerably in recent years worldwide. Regardless of whether or not lifestyle choices and habits during the autonomic life contribute clearly to these epidemics, there is growing evidence suggesting that the maternal nutritional environment during critical stages of *in utero* development programs a higher risk of metabolic diseases later in life [3]. Offspring of diabetic pregnancy (ODP) are at risk for development of obesity and abnormal glucose metabolism during childhood, adolescence, and adulthood [4]. Thus, in addition to increased risk for the development of vascular disease in later life [5], obesity has been included as an outcome of interest in the offspring born to pregnancies complicated by diabetes [6]. Indeed, it is well known that fetal overgrowth has been related to increased transplacental transfer of glucose, stimulating the release of insulin by the fetal beta cell and subsequent macrosomia. Optimal maternal glucose control was shown to decrease perinatal mortality and morbidity, but whether or not improved glycemic control decreases the risk of obesity later in life is yet to be concluded. The recent decades are characterized by a large increase in maternal obesity and subsequently gestational diabetes mellitus and Type 2 diabetes [7]. From the Helsinki Birth Cohort Study, it was shown that maternal BMI (body mass index) directly correlates with health outcomes in infants, particularly cardiovascular disease and Type 2 diabetes [8]. Moreover, the Generation R Study demonstrated that higher weight gain in early pregnancy was associated with an adverse cardio-metabolic profile in infants, particularly mediated by childhood adiposity [9]. These results were confirmed through the Viva Project, a Boston-area prebirth cohort, where infants born to heavier mothers were shown to have more overall and central fat and greater cardio-metabolic risk, while infants of women with higher gestational weight gain had greater adiposity and higher leptin [10]. The worst outcomes are usually observed when nutritional conditions during gestation that have induced adaptive responses in the fetus and neonate, differ from consecutive postnatal nutrition (referred to as “nutritional mismatch” between early *in utero* life and adulthood) [11,12]. Metabolic diseases such as obesity, Type 2 diabetes and hypertension are the main diseases studied in the context of DOHaD, but many other functions are affected. Thus, in humans, early “conditioning processes” [13] were shown also to affect, among others, bones [14], psychiatric health [15] or fertility [16].

Most data in humans are based on epidemiological evidences from large cohort studies. The exploration of mechanisms leading to DOHaD and possible nutritional corrections during pregnancy or in the early neonatal period to reverse an inadequate programming can be achieved using animal models. Understanding key similarities, differences and limitations of various animal models used to study DOHaD is critical for translating animal findings to humans. Particular interest is given to the placentation [17] as the placenta is a key element for conveying information on maternal metabolism to the fetus [18,19,20].

## 2. Choice of Animal Models

As a consequence of obvious ethical considerations concerning what is feasible or not in the field of human biomedical research, animal models are of critical importance. The choice of one animal model over another is important and will depend on its similarity to the human being in terms of anatomy, physiology and metabolism, but also length of gestation, litter size, weight at birth, which is essential for inferring relevant information for possible application to the clinical context (Table 1). Animal models are particularly helpful for studies in the field of fetal programming (or DOHaD); indeed they allow us to clarify the critical windows of development, with exposure windows ranging from the preconception period and/or implantation until the postnatal period (breastfeeding), alone or altogether or through nutritional mismatch. They also allow the study of the effects of different types of maternal conditions (*in utero* exposures) such as under, over- and malnutrition, and/or maternal metabolic status (obesity and/or diabetes), among others (food pollutants, maternal stress…).

Placentation in humans is classified as hemochorial, *i.e.*, the trophoblast is in direct contact with the maternal blood. Human trophoblast is considered the most invasive of all hemochorial placentas with very deep invasion of the maternal uterine wall to reach the maternal arteries. Direct contact with maternal blood, however, only occurs at the end of the first trimester. Non-human primates, rodents and lagomorphs (rabbits) also possess a hemochorial placenta. In the murine model, however, the placenta initially derives from the yolk sack and its definitive placentation only occurs in mid-gestation (total gestation time: 19–20 days) with a very limited invasion [21]. Other model species, such as sheep or pigs, possess an epitheliochorial placenta, which means that the placenta is just apposed on the maternal endometrium, with a concomitant thinning of the six trophoblastic and endometrial cell layers and extensive interdigitation of maternal and fetal tissues, which largely increase exchange surface.

Mice are the most commonly used animal models worldwide, providing a low cost, easy to handle, ethically acceptable option. They also provide ample access to genomic tools and transgenic lines. The discoid hemotrichorial placentation and the developmental and epigenetic processes are well described in these species at all stages of gestation. Limitations of this model include its small size, large number of embryos per litter and very short length of gestation (18–19 days), making it difficult to perform *in vivo* imaging and longitudinal sampling, and study chronic perturbations at specific stages of gestation. Given recent developments allowing genetic modifications in non-rodent models, additional animal models are increasing in popularity [22,23,24]. Moreover, their anatomical and physiological properties present advantages that can justify their use even without the use of genetic modifications, and ample knowledge and tools are becoming increasingly available [25].

Rabbits have been long used as animal models in toxicology. Their lipid metabolism is also closer to that of humans than mice [26]. Rabbits are particularly suitable for studies on the blastocyst because of their large size, allowing embryonic genome analyses in single embryos and easy analysis of gastrulation stages (because gastrulation begins before implantation in the rabbit) [27,28]. Their placental structure (discoid hemodichorial as opposed to hemotrichorial in mice) is closest to that of humans (discoid hemodichorial at early stages and hemomonochorial in late pregnancy) [29,30]. The adult size of rabbits enables the use of ultrasound imaging techniques for the monitoring of fetal development as in pregnant women [30,31]. It is a very docile species that reproduces easily with relatively short reproductive cycles and a lifetime that is long enough for exploring the occurrence of chronic diseases in offspring. Limitations of this model include the fact that it is a polytocous (several young rabbits per litter) species and the fragility of the offspring litter around weaning.

Sheep are human-sized animals with a long gestational period (five months) and usually a monotocous species (mostly one or two lambs per litter) as in humans. At term, the lamb weighs about the same as a human fetus and this has contributed to the popularity of this model in obstetrical research [30]. Sheep are easy to handle and pregnant animals tolerate invasive procedures aimed at reducing or evaluating fetal responses to different environments. Therefore sophisticated techniques have been developed for functional studies *in utero* after full recovery from anesthesia and surgery [18,32]. As a result, the available knowledge on fetal physiology is greater for sheep than any other species. The main disadvantages of this model are that sheep have a very different placentation (cotyledonary-type epitheliochorial placenta) than humans and also that they are ruminants with a very different digestive physiology and lower glycaemia than monogastric species. They are also expensive and housing, care and use of sheep is highly regulated by government agencies such as in the U.S. and Europe.

Pigs are considered to be one of the best domestic animal models for human nutrition due to their omnivorous diet, and are widely used because of the close resemblance between their gastro-intestinal tract and that of humans. Pigs are excellent for studying the environment of early stages of fertilization and early development. Their size makes sample preparations for proteomic analysis very easy. Limitations of this model include the fact that it is a polycotous species, with a diffuse epitheliochorial placenta that is different from humans. Their breeding is also costly and the adult animals are difficult to handle. Although their placentation is very different from that of humans, pigs remain a good post-natal model for intrauterine growth retardation (IUGR) as this condition occurs spontaneously in most litters, so that full siblings can be used as controls.

Non-human primates could be considered as the gold standard due to their important similarities to humans. However, their use for biomedical research is greatly limited by their behavioral and social organization, raising important ethical questions, as well as their elevated cost.

Through animal models, we can observe the subsequent long-term phenotypes of offspring (physiological, cardio-metabolic and behavioral characteristics) and explain the molecular mechanisms by transversal and multidisciplinary approaches. The most common species used in this field are rodents, as well as rabbits (lagomorphs), sheep, pigs and non-human primates, depending on the experimental approaches and tools used to observe in offspring the effects of maternal conditions.

## 3. Maternal Conditions Associated with DOHaD

**Figure 1:**
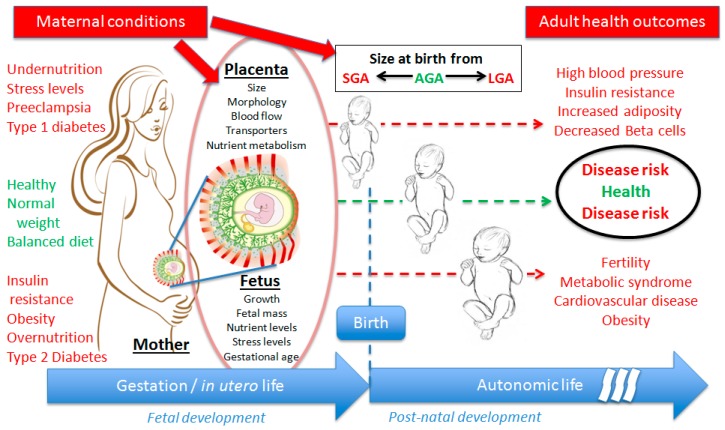
Schematic representation of the DOHaD (developmental origins of health and diseases) concept in human beings, showing the effects of several maternal conditions (non exhaustive) during the gestation on feto-placental unit development (affected parameters), leading to particular birth size from SGA (small for gestational age) to LGA (large for gestational age), and the most observed outcomes in terms of health at adulthood.

It is now well-established from epidemiological studies that both early nutrition and maternal metabolic status during pregnancy play a seminal role in determining the offspring’s long-term health and metabolism. These maternal conditions affect the growth trajectory and the function of the feto-placental unit, leading to various birth sizes from small (SGA) to large for gestational age (LGA), correlated to different adverse health outcomes at adulthood (Figure 1).

### 3.1. Placental Insufficiency

**Figure 2:**
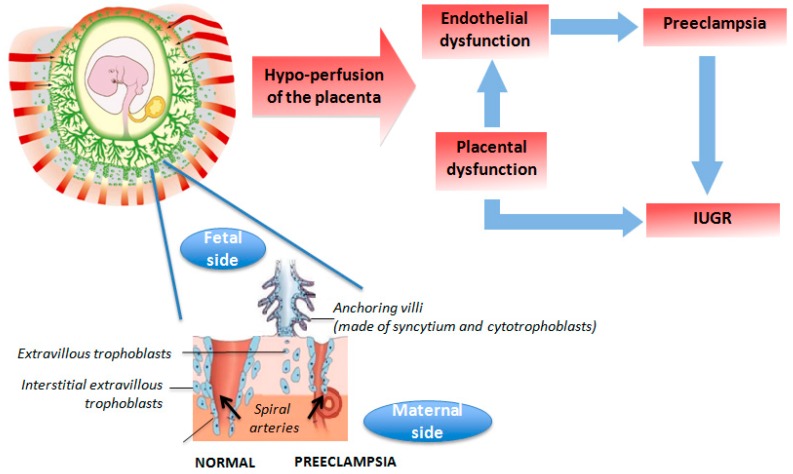
Schematic representation of materno-fetal vascularization in human normal pregnancy or preeclampsia conditions leading to intrauterine growth retardation (IUGR).

In humans in westernized countries, IUGR is rarely the consequence of malnutrition but is rather due to placental insufficiency due to abnormal placental vascularization (such as preeclampsia or pregnancy-induced hypertension) [33]. In preeclampsia, a disease specific to humans with no known equivalent syndrome in animals but for some non-human primate species, spiral arteries do not undergo physiological remodeling into the myometrial segment through trophoblast invasion, which explains the utero-placental ischemia and inflammatory response (Figure 2). Animal models of surgically-induced placental vascular restriction remain unsatisfactory [34]. Nevertheless, the use of spontaneous or less invasively-induced models of IUGR of vascular origin in pigs or sheep has demonstrated that the same pattern of long-term effects were observed as for maternal undernutrition [35].

### 3.2. Maternal Malnutrition

Although maternal malnutrition is not the most common cause of fetal undernutrition, nutrition during pregnancy plays a significant role in both fuel supply to the fetus and placentation. Vasculogenesis and angiogenesis are key events for the development of the placenta. Placental vascularization progresses throughout gestation and is controlled by both physical and chemical factors such as oxygen and growth factors [36,37]. The maternal environment is able to modulate placental vascularization and alter the transport of respiratory oxygen and nutrients. For example, in ewes underfed during gestation, the placental expression of vascular endothelial growth factor (VEGF) receptor transcripts was reduced by 50% on day 130 compared to ewes that received standard diet, while VEGF transcripts remained unchanged [38]. Placental circulation, which is central to uterine and umbilical blood flows, is therefore an important player for the success of pregnancy.

**Figure 3:**
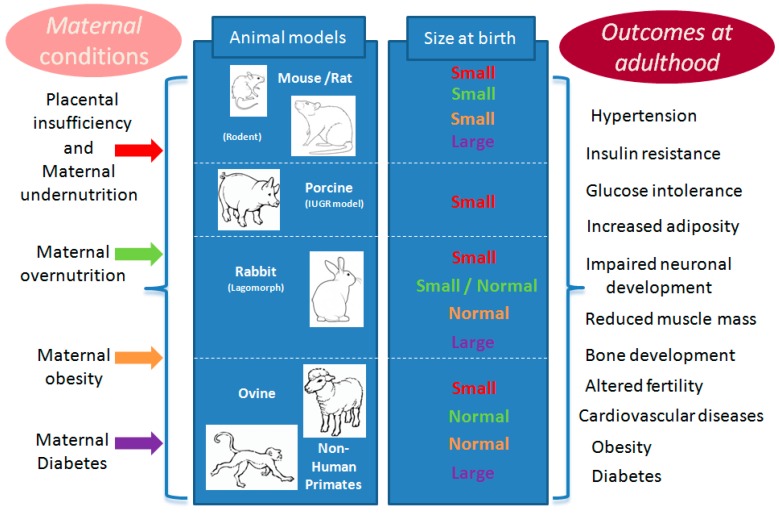
Schematic representation of prevalent models for studying exposures to maternal nutrition or metabolism during gestation, the consecutive and species-specific birth size, and the usual outcomes in terms of health for all animal models at adulthood. One color is attributed to each kind of exposure: **red** for placental insufficiency and maternal malnutrition (undernutrition), **green** for over nutrition, **orange** for obesity and **purple** for diabetes. The column “size at birth” gave the fetal response to each kind of maternal conditions in term of size compared to control conditions in the same species. The same color is used for the “small” or “normal” or “large” response to each corresponding maternal conditions compared to normal (or expected) size for same age in the same species.

In rodents, most experiments that induce global undernutrition or protein restriction during gestation, lead to fetal IUGR with subsequent increase in the incidence of hypertension, excess adipose tissue deposition, abnormal glucose metabolism and dyslipidemia in adult offspring [39] (Figure 3). In mice, a reduction in longevity of 32% was observed in male offspring born to females submitted to a low protein diet during gestation and fed a high fat/high carbohydrates diet after weaning compared to controls fed a normal diet [40]. In the same study, some of the dams were fed a low protein diet during lactation that slowed down post-natal growth. Thus, offspring born to dams fed normally during gestation and with reduced neonatal growth had the longest lifespan, underlining the importance of the immediate post-natal nutrition for conditioning post-natal phenotype [40]. Similar observations in several model species have confirmed the robustness of these phenomena in mammals [41,42].

These effects, however, are most often only observed in adult animals and so caution should be taken when interpreting growth outcomes from studies limited to the neonatal period or infancy. In general, prolonged and relatively severe maternal undernutrition is necessary to affect birth weight and post-natal growth in sheep [43] and pigs [44]. Undernutrition at the beginning of gestation, however, can affect offspring body composition and survival at birth with or without affecting birth weight *per se*, but also adult metabolism, production and body composition. As for examples of the latter, increased adiposity is often observed [45]. Moreover, increased glucocorticoid sensitivity was observed in sheep after maternal undernutrition in early pregnancy [46]. In another experiment, maternal undernutrition of Large White sows from mating to 50 days of pregnancy did not affect body weight, lean tissue and adipose tissue yield in offspring, whereas the composition of muscle in terms of myofiber type was slightly affected [47]. A maternal protein restriction, however, was shown to reduce the lean and increase the fat contents of six-month old offspring with a tendency for reduced number of muscle myofibers associated with reduced expression of IGF2 mRNA (messenger RNA of *Insulin-like growth factor 2* gene) [48].

### 3.3. Maternal Overnutrition and/or Obesity

Although one would expect that maternal overnutrition would always result in fetal overgrowth, but this is not always the case. As a matter of fact, in many species, maternal overnutrition or obesity results in IUGR through mechanisms that affect blood flow to the placenta [49]. For example, over-nourished pregnant young sheep are characterized by an increase in umbilical artery Doppler indices at mid-gestation, which precede the reduction in fetal growth velocity [49]. Feeding an obesogenic diet before breeding and throughout gestation and lactation was shown to induce obesity, hyperglycaemia and hyperinsulinemia in female rats, which induced subsequent insulin resistance in their male offspring [50,51] (Figure 3). Similar effects were observed in rabbits [52]. Dietary-induced obesity gives rise to placental inflammation at term. Indeed, this placental inflammation has been observed earlier in pregnancy in a sheep model of overfeeding-induced obesity and was associated with significant fetal consequences including higher plasma content of free fatty acids, triglycerides and cholesterol [53]. In another sheep study, maternal obesity increased collagen content and cross-linking in offspring muscle, which might be partially due to reduced collagen remodeling, favoring insulin resistance [54]. Moreover, when female rats were fed an obesogenic diet before conception but not during pregnancy, maternal treatment also enhanced offspring response to a high fat diet [51]. These data demonstrate that maternal obesity conditions the offspring to obesity, even if nutritional intake is controlled during pregnancy. More recent studies also indicate that not only metabolic disorders are observed in offspring, but also behavioral abnormalities such as hyperactivity [55]. A study in non-human primates showed that maternal dietary-induced obesity caused epigenetic modifications in offspring hepatic genes that could alter metabolism and predispose them to obesity later in life [56]. In adult ewes, dietary-induced obesity was associated with offspring macrosomia [57], hyperglycemia and hyperinsulinemia with markedly increased pancreatic weight and beta cell numbers [58]. Maternal obesity was also shown to downregulate fetal myogenesis at mid-gestation through the Wnt/beta-catenin signaling pathway (Wnt are secreted glycolipoproteins via the transcription co-activator beta-catenin) [59]. As demonstrated by these animal studies, maternal overnutrition affects offspring body composition through multiple mechanisms, requiring a multi-faceted approach for the prevention of developmentally programmed obesity.

### 3.4. Maternal Diabetes

Models of maternal diabetes are associated with various degrees of pancreatic dysfunction and insulin resistance and thus there is a large phenotypic variation in offspring, as described in human medicine. As an example, streptozotocin injection in female rats during gestation, inducing the destruction of pancreatic beta cells, is associated with fetal macrosomia and subsequent hyperglycemia and hyperinsulinemia in offspring [60]. Conversely in alloxan-induced diabetic rabbits, the birth weights of fetuses from severely diabetic rabbit does were only slightly lower than those of controls, whereas offspring from mildly diabetic does had birth weights that were significantly higher than controls [61] (Figure 3). Still, in alloxan-induced diabetic rabbits, maternal hyperglycemia during the pre-implantation period is correlated with an increase in advanced glycation end-product formation (oxidative stress) in the uterine environment and the embryo itself, compromising further development and health outcomes [62]. When glucose is infused in rats in late gestation, this provokes hyperglycemia-induced disorders in insulin secretion in offspring [63,64]. Similarly, in sheep, while diabetes does not occur naturally and is not chemically-induced, an artificial transient maternal hyperglycemia in the last third of gestation can be used to explore the effect on fetal growth (macrosomia) and further offspring health outcomes [65].

Finally, transgenic pigs have been developed to provide systemic insights into developmental consequences of maternal hyperglycemia when important issues of early human development cannot be adequately simulated in mouse models [66]; they exhibited decreased beta cell mass and lower fasting insulin levels, with a stable diabetic phenotype compared with littermate controls [67]. These models will be very useful to study the effects of maternal diabetes on offspring development.

## 4. Interventional Experiments for Remediation of Long Term Effects

As summarized through some examples above, there is rapidly emerging evidence regarding the adverse effects of maternal obesity and diabetes in the DOHaD in both humans and animal models. There is a strong need for implementing prenatal and postnatal prevention strategies. Various approaches have been tested and several are highlighted below.

In humans, prevention strategies are the basis of the 1000 days initiative, which aims to improve nutrition recommendations for mothers and children from the beginning of woman’s pregnancy until the second birthday of her child [68].

Switching obese rats from an obesogenic to a standard diet one month before breeding and maintaining this diet until delivery considerably improved adult offspring outcomes [69]. Similar results were obtained in pregnant obese ewes that were returned to a diet according to their nutritional requirements early in gestation [69]. In another attempt in mice, increasing the n-3/n-6 polyunsaturated fatty acid (PUFA) ratio of maternal plasma reduced the consequences of maternal obesity-associated inflammation during pregnancy and prevented adverse fetal metabolic outcomes [70]. The effects of exercise before and throughout gestation in obese female rats have been tested on offspring outcome [69]: maternal exercise in obese rats decreased body weight, fat and adiposity indices in male offspring at postnatal day 650. Moreover, exercise also improved the adverse metabolic outcomes in offspring.

There is ample evidence in animal models that the periconceptional environment plays an important role in conditioning the offspring’s post-natal health [71,72,73,74,75]. Thus, attempts to correct the nutritional environment of the mother before conception have been performed. The response to an adverse maternal nutritional environment depends on the ability of the embryo to adapt itself to such an environment, which is acquired before implantation, as demonstrated by studies in mice submitted either to low protein [75] or high fat diets [76]. Thus, correcting nutritional imbalance in women before the establishment of pregnancy might be included in the strategies to prevent child and adult obesity. Moreover, still in the periconceptional context, recent data in mouse models indicate that the father’s diet and/or metabolic condition (dyslipidemia, glucose intolerance, and obesity) can affect epigenetic marks in the spermatozoa and induce altered metabolism in offspring [77,78,79,80]. This indicates that not only the woman, but also her male partner need to be considered when trying to prevent childhood obesity before conception [81].

## 5. Conclusions

In conclusion, the maternal conditions experienced during *in utero* life lead to adaptive changes of structure and function of placenta and of vital organs in the fetus. Most of the experimental studies in fetal programming have been driven to better characterize the impact of maternal conditions during gestation on offspring health from the prenatal stage (*in utero* life) to adulthood. Most of these effects are induced very precociously in life and may involve epigenetic processes (but not only such processes). The long-term health outcomes in offspring very often include insulin resistance, excess adiposity and cardio-metabolic disorders, whatever the studied species (rats, guinea pigs, mice, rabbit, sheep, pigs, and non-human primates). Research on DOHaD should now be intensified to identify the mechanisms leading to the observed effects in adulthood.

Animal models are essential to the understanding of these mechanisms and the development of interventional therapies before they are implemented in humans.

Remediating long-term consequences might be possible using pre-conceptional, gestational or perinatal interventions. These strategies have been proven effective in the past as shown using folic acid and n-3 PUFA that have been used to improve the metabolic or inflammatory profile of the mother (from the preconception period to the weaning of the offspring).

All remediation efforts need to be challenged to evaluate mechanisms leading to both short and long-term effects including functional adaptability, susceptibility, and reversibility. In doing so, it is important to remember that non-rodent animals should not be neglected, as they are often more physiologically-appropriate models to human conditions. This review highlights specific maternal, placental and fetal characteristics associated with DOHaD and provides guidance in the selection of appropriate animal models to understand and prevent developmentally programmed disease.

## Figures and Tables

**Table 1:** Pertinence of animal models and availability of tools classified from − (very little advantages) to ++++ (very advantageous or largely available). ?: no (or very few) data available; NA: not applicable. It must be noted that with the development of genome editing in mammalian species, transgenic models are quickly becoming easily available and reasonably priced in non-rodent animals.

Criteria	Mice	Rats	Rabbits	Pigs	Ruminants	Non-Human Primates	Comments
Cost	++++		+++	+	+	-	The cost of mice being lower, more groups can be developed.
Nutrition	+++	+++	+	++++	−	++++	Pigs have a digestive tract very similar to humans. Mice and rats can tolerate high fat diets. Pig and rabbit lipid metabolism are close to humans.
Pre-implantation development	+++	?	+++	?	++	++	The embryonic genome activation takes place at the 8–16 cell stages in all species, including humans, but for mice (2 cell stage). Mice are the best studied for preimplantation development.
Blastocyst stage	+++		+++	++	++	NA	Detailed knowledge of mice development is available. Rabbit blastocysts are used for fine analysis of gastrulation. They yield enough cells for individual embryo analysis.
Placental physiology	+++		+++		++	++++	Primates, rabbits and rodents possess a hemochorial placentation (rabbit placenta being closest to humans). Ruminant and pig placenta are different.
Fetal development	++		++	++	+++	++++	Polycotous species are less representative for human development compared to monocotous species.
Management	++++	++++	++++	++	+++	++	Small species are highly manageable, with short intergenerational intervals. In larger animals, ultrasound imaging of fetal growth is easily performed.
Lactation	++	+++	++++	++++	+++	+++	In rabbits, suckling occurs only once a day, the study of milk intake and milk production easy. The rat “pup in the cup” model exists only in the rat.
Acceptability	++++	++++	++++	+++	+++	+/-	Mice and rats are the most acceptable.
Genomic tools	++++	+++	++	+++	+++	+++	Genomic and epigenetic tools and antibodies remain most available in mice, although possibilities increase in other species.
Transgenic models	++++	-	++	++	+	−	Transgenic mice models are largely available but transgenic models are also available in rabbits and pigs.
Programming outcomes that are identified through the measurements of physiological parameters in the offspring							
Overweight	yes	yes	yes	yes	yes	yes	The ruminants have a different glucose metabolism from monogastric species and do not become diabetic.
Hypertension	yes	yes	yes	yes	yes	yes	
Diabetes	yes	yes	yes	yes		yes	
Behavior	yes	yes			yes	yes	
